# Scleral buckle infections: microbiological spectrum and antimicrobial susceptibility

**DOI:** 10.1186/1869-5760-3-67

**Published:** 2013-12-13

**Authors:** Jay Chhablani, Sameera Nayak, Animesh Jindal, Swapna R Motukupally, Annie Mathai, Subhadra Jalali, Rajiv Reddy Pappuru, Savitri Sharma, Taraprasad Das, Harry W Flynn, Avinash Pathengay

**Affiliations:** 1 Srimati Kanuri Santhamma Centre for Vitreo-Retinal Diseases, Kallam Anji Reddy Campus, L. V. Prasad Eye Institute, Hyderabad 500 034, India; 2 Vitreo-Retina services. G. M. R. Varalakshmi Campus, L. V. Prasad Eye Institute, Visakhapatnam 530 040, India; 3 Jhaveri Microbiology Centre, Prof. Brien Holden Eye Research Centre, Kallam Anji Reddy Campus, L.V., Prasad Eye Institute, Banjara Hills, Hyderabad 500 034, India; 4 Bascom Palmer Eye Institute, Department of Ophthalmology, University of Miami, Miller School of Medicine, Miami, FL 33136, USA

**Keywords:** Buckle infection, Scleral buckle, Buckle, Sponge

## Abstract

**Background:**

The purpose of the present study was to evaluate the microbiological spectrum and antimicrobial susceptibility in patients with scleral buckle infection. Medical records of all the patients diagnosed as buckle infection at L. V. Prasad Eye Institute between July 1992 and June 2012 were reviewed in this non-comparative, consecutive, retrospective case series.

**Findings:**

A total of 132 eyes of 132 patients underwent buckle explantation for buckle infection during the study period. The incidence of buckle infection at our institute during the study period was 0.2% (31 out of 15,022). A total of 124 isolates were identified from 102 positive cultures. The most common etiological agent isolated was *Staphylococcus epidermidis* (27/124, 21.77%) followed by *Mycobacterium* sp. (20/124, 16.13%) and *Corynebacterium* sp. (13/124, 10.48%). The most common gram negative bacilli identified was *Pseudomonas aeruginosa* (9/124, 7.26%). The median interval between scleral buckling surgery and onset of symptoms of local infection was 30 days. All eyes underwent buckle explantation and median time interval between primary SB surgery and explantation was 13 months. Recurrent retinal detachment was observed in two cases at 7 and 48 months, respectively, after buckle explantation. Gram positive, gram negative, and acid-fast organisms isolated from 2003 to 2012 were most commonly susceptible to vancomycin (100%), ciprofloxacin (100%), and amikacin (89%). Susceptibility to ciprofloxacin during the same time period was observed in 75% (15/20), 100% (13/13), and 87% (7/8) of gram positive, gram negative, and acid-fast isolates, respectively.

**Conclusion:**

Scleral buckle infection is relatively rare and has a delayed clinical presentation. It is most commonly caused by gram positive cocci. Based on the current antimicrobial susceptibility, ciprofloxacin can be used as empirical therapy in the management of scleral buckle infections.

## Findings

### Background

Scleral buckle (SB)-related complications include induced myopia, diplopia, foreign body sensation, infection, extrusion, and intrusion [1-5]. There are several reports on explantation of SB for various indications [1,4,6-13]. Buckle infection is one of the common causes for buckle explantation. The reported incidence of buckle infection varies from 0.5% to 5.6% [5,8,14-16]. Buckle infection may lead to severe complications like endophthalmitis and panophthalmitis [2]. The literature about buckle infection includes mostly case reports and small case series. We performed a retrospective analysis of patients who underwent SB removal for SB infection and studied their microbiological spectrum and antimicrobial susceptibility.

### Methods

After obtaining Institutional Review Board approval, a retrospective chart review of all subjects with scleral buckle infection was performed. Collected data included demographic profile, microbiology culture, and antibiotic susceptibility of isolates determined by Kirby Bauer disc diffusion method, management, duration since primary SB surgery was performed and incidence of RD after buckle removal with outcome after re-surgery for RD. SB explanted for diplopia, strabismus, and retinopathy of prematurity were excluded.

### Results

Between July 1992 and June 2012, a total of 15,022 SB surgeries were performed at our center. Scleral buckle explantation was carried out in 132 eyes of 132 patients out of whom 31 patients had undergone primary buckle surgery at our institute, making the incidence of buckle infection at our institute to be 0.2% (31 out of 15,022).

#### Demographic profile

Out of 132 patients, 101 were males and 31 were females. The mean age at diagnosis of buckle infection was 43.6 years, ranging from 4 to 80 years. Right eye was involved in 65 patients. The median follow-up in the study cohort was 12 months, ranging from 1 month to 16 years. Median duration of symptoms before presentation was 30 days, ranging from 1 day to 5 years. The most common symptoms were pain (84/132, 63.64%), redness (46/132, 34.85%), watering (45/132, 34.09%), and foreign body sensation (34/132, 25.76%). Thirty four (25.76%) patients complained of diminution of vision. Most common clinical presentation was diffuse or localized conjunctival congestion associated with chemosis in 109 (82.57%) eyes followed by buckle/suture exposure in 108 (81.82%) eyes and lid edema and purulent discharge in 44 (33.33%) eyes. One hundred out of 132 (75.7%) eyes had solid buckle exposure, five (3.79%) eyes had only suture exposure, and three (2.27%) eyes had exposed silicone sponge.

All patients underwent buckle explantation. Median time interval between primary SB surgery and explantation was 13 months (1 week to 16 years). Three eyes presented as panophthalmitis and underwent evisceration along with buckle explantation. Two eyes presented as buckle infection with endophthalmitis, one eye underwent vitreous biopsy and intraocular antibiotics and the other underwent pars plana vitrectomy with intraocular antibiotic injection after buckle explantation. Endophthalmitis resolved in both the eyes. Only two eyes developed recurrent retinal detachment after buckle explantation after 7 and 48 months, respectively. Both underwent vitreo-retinal surgery with successful anatomical outcome.

#### Microbiological spectrum

Microbiological analysis results were available for 126 patients, and 102 (80.95%) had culture positivity during the entire study period. Our group previously published microbiological spectrum and susceptibility data from 1992 to 2002 [17]. From 2003 to 2012, 66 patients underwent buckle explantation for infection. Microbiological data was available for 60 patients and 47 (78.3%) samples were culture positive. Fifty-one isolates were identified from these 47 culture positive buckles. Four cases had polymicrobial infection. Gram positive cocci (GPC), gram negative bacilli (GNB), gram positive bacilli (GPB), fungi, and acid-fast bacilli were identified in 14 (27%), 13 (25%), 7 (14%), 9 (18%), and 8 (16%) isolates, respectively. During the total study duration from 1992 to 2012, the most common organism isolated was *Staphlycoccus epidermidis* (27/124, 21.77%) followed by *Mycobacterium* sp. (20/124, 16.13%) and *Corynebacterium* sp. (13/124, 10.48%). The most common gram negative bacilli identified was *Pseudomonas aeruginosa* (9/124, 7.26%). This data is detailed in Table 1.

**Table 1:** Isolated organism and their antibiotic susceptibility in eyes with buckle infection

	**No. /% of isolates (1992 to 2002) **[17]	**No. /% of isolates (2003 to 2012)**	**Percentage of organisms susceptible to different antibiotics (2003 to 2012)**						
Total isolates	73	51	A	C	CE	CH	CI	G	V
Total bacteria	62/84.9%	43/84%							
Gram positive cocci	30/41.1%	14/27%	100 (*n* = 3)	93 (*n* = 14)	nd	82 (*n* = 11)	85 (*n* = 13)	77 (*n* = 13)	100 (*n* = 13)
*Staphylococcus epidermidis*	20/27.4%	7/14%	100 (*n* = 1)	86 (*n* = 7)	nd	67 (*n* = 6)	67 (*n* = 6)	83 (*n* = 6)	100 (*n* = 5)
*Staphylococcus aureus*	6/8.2%	2/4%	100 (*n* = 1)	100 (*n* = 2)	nd	100 (*n* = 2)	100 (*n* = 2)	100 (*n* = 2)	100 (*n* = 2)
*Streptococcus pneumoniae*	4/5.5%	1/2%	nd	100 (*n* = 1)	nd	100 (*n* = 1)	100 (*n* = 1)	nd	100 (*n* = 1)
*Brevibacterium* species	-	1/2%	100 (*n* = 1)	100 (*n* = 1)	nd	100 (*n* = 1)	100 (*n* = 1)	100 (*n* = 1)	100 (*n* = 1)
Only gram positive cocci (no species identified)	-	3/6%	nd	100 (*n* = 3)	nd	100 (*n* = 1)	100 (*n* = 3)	33 (*n* = 3)	100 (*n* = 3)
Gram negative bacteria	7/9.6%	13/25%	100 (*n* = 9)	0 (*n* = 6)	60 (*n* = 5)	46 (*n* = 13)	100 (*n* = 13)	69 (*n* = 13)	40 (*n* = 5)
*Pseudomonas aeruginosa*	-	5/10%	100 (*n* = 3)	0 (*n* = 2)	67 (*n* = 3)	0 (*n* = 5)	100 (*n* = 5)	60 (*n* = 5)	0 (*n* = 1)
*Neisseria* species	-	2/4%	100 (*n* = 1)	0 (*n* = 1)	100 (*n* = 1)	100 (*n* = 2)	100 (*n* = 2)	50 (*n* = 2)	100 (n = 1)
*Pseudomonas* species	2/2.7%	2/4%	100 (*n* = 1)	0 (*n* = 1)	nd	50 (*n* = 2)	100 (*n* = 2)	100 (*n* = 2)	nd
*Aeromonas hydrophila*	-	1/2%	nd	0 (*n* = 1)	nd	100 (*n* = 1)	100 (*n* = 1)	100 (*n* = 1)	0 (*n* = 1)
*Acinetobacter baumannii*	2/2.7%	1/2%	nd	0 (*n* = 1)	nd	0 (*n* = 1)	100 (*n* = 1)	0 (*n* = 1)	0 (*n* = 1)
*Ralstonia pickettii*	-	1/2%	100 (*n* = 1)	nd	0 (*n* = 1)	0 (*n* = 1)	100 (*n* = 1)	100 (*n* = 1)	nd
Unidentified gram negative bacilli	-	1/2%	nd	nd	nd	100 (*n* = 1)	100 (*n* = 1)	0 (*n* = 1)	100 (*n* = 1)
Gram positive bacilli	10/13.7%	7/14%	100 (*n* = 2)	86 (*n* = 7)	nd	57 (*n* = 7)	57 (*n* = 7)	86 (*n* = 7)	100 (*n* = 6)
*Corynebacterium* species	6/8.2%	7/14%	100 (*n* = 2)	86 (*n* = 7)	nd	57 (*n* = 7)	57 (*n* = 7)	86 (*n* = 7)	100 (*n* = 6)
Acid-fast organism	15/20.5%	9/18%	89 (*n* = 9)	22.22 (*n* = 9)	0 (*n* = 4)	33.3 (*n* = 9)	87 (*n* = 8)	89 (*n* = 9)	33.3 (*n* = 9)
*Mycobacterium chelonae*	6/8.2%	6/12%	83 (*n* = 6)	33 (*n* = 6)	0 (*n* = 3)	33.3 (*n* = 6)	80 (*n* = 5)	83 (*n* = 6)	33 (*n* = 6)
*Mycobacterium fortuitum*	6/8.2%	2/4%	100 (*n* = 2)	0 (*n* = 2)	0 (*n* = 1)	0 (*n* = 2)	100 (*n* = 2)	100 (*n* = 2)	50 (*n* = 2)
*Nocardia asteroides*	3/ 4.1%	1/2%	100 (*n* = 1)	0 (*n* = 1)	nd	100 (*n* = 1)	100 (*n* = 1)	100 (*n* = 1)	0 (*n* = 1)
Fungi	11/15.1%	8/16%	nd	nd	nd	nd	nd	nd	nd
*Aspergillus flavus*	4/5.5%	4/8%	nd	nd	nd	nd	nd	nd	nd
*Aspergillus terreus*	3/ 4.1%	1/2%	nd	nd	nd	nd	nd	nd	nd
Dematiceous fungus	-	1/2%	nd	nd	nd	nd	nd	nd	nd
*Acremonium* species	-	1/2%	nd	nd	nd	nd	nd	nd	nd
*Curvularia lunata*	-	1/2%	nd	nd	nd	nd	nd	nd	nd

A, amikacin; C, cefazolin; CE, ceftazidime; CH, chloramphenicol; CI, ciprofloxacin; G, gentamicin; V, vancomycin; *n*, number of isolates for which susceptibility was checked for a particular antibiotic, nd, not done.

#### Antimicrobial susceptibility

Details about the organisms and their susceptibilities from 2003 to 2012 are presented in Table 2. Both gram positive cocci and bacilli were most susceptible to vancomycin (100%). The susceptibility to ciprofloxacin was 85% and 57% for gram positive cocci and gram positive bacilli, respectively. Gram negative bacilli were most susceptible to ciprofloxacin and amikacin (100%) followed by gatifloxacin (69%) and ceftazidime (60%). Acid-fast bacilli were most susceptible to amikacin and gatifloxacin (89%) followed by ciprofloxacin (87%).

**Table 2:** Comparison of antibiotic sensitivity with previous report from the same institute

**Organism sensitive to antibiotic**	**2003 to 2012**	**1992 to 2002 **[17]
GPC sensitive to cefazolin	93%	86.7%
GPC sensitive to vancomycin	100%	93.1%
GPC sensitive to ciprofloxacin	85%	73.3%
GNB sensitive to amikacin	100%	14.3%
GNB sensitive to ciprofloxacin	100%	85.7%
GPB sensitive to cefazolin	86%	85.7%
GPB sensitive to gentamicin	86%	100%
GPB sensitive to vancomycin	100%	80%
AF sensitive to amikacin	89%	80%
AF sensitive to gentamicin	89%	56.2%
AF sensitive to ciprofloxacin	87%	30.8%

GPC, gram positive cocci; GNB, gram negative bacilli; GPB, gram positive bacilli; AF, acid-fast stain positive.

### Discussion

In the current study, SB infection is reported in solid silicone explants in contrast to previous studies which were mainly based on silicone sponge explants [1,18,19]. The probable reason for this difference is the decline in usage of sponges in the last few decades. The scleral buckle infection rate was 0.2% compared to 0.5% to 5.6% in published literature [5,8,14-16].

It is interesting to note that 18.2% (24/132) of eyes had buckle infection without any buckle/suture exposure. The probable source of infection in such eyes could be organism gaining entry during the surgery and causing a biofilm formation as reported in earlier studies [20]. Biofilm has been demonstrated on the surfaces and ends of solid silicon elements. Ability of biofilm to withstand antimicrobial treatment can lead to persistence of scleral buckle infections [21].

As per the previous reports, 70% to 82% re-detachment of retina occurred within 90 to 180 days following SB removal [9]. In the current study cohort, only two eyes developed retinal detachment after buckle removal, at 7 and 48 months following SB removal. Mean follow-up period was 39.84 months with a re-detachment rate of 1.51% which is less than previous studies [1,4,9,12,22]. In our study cohort, only 33.33% SB removal were performed within 6 months of buckle surgery whereas in majority cases (66.67%), SB removal was performed after 6 months of buckle surgery. These factors could influence the low incidence of retinal detachment rate observed in the current study.

In contrast to a study by Wirostko et al. [23], which reported culture positivity of 35%, the current study reports 80.95% culture positivity among the buckles explanted for infection. Compared to the previous report [17], though GPC remained the most common bacteria to cause buckle infection, we observed an increase in GNB isolates (Table 1). The percentage of GNB isolated from 1992 to 2002 was 9.6% (95% confidence interval = 4.72% to 18.5%) and that from 2003 to 2012 was 25% (95% confidence interval = 15.5% to 38.8%) but the difference is statistically not significant as there is an overlap of the 95% confidence intervals. The percentage of acid-fast bacilli and fungi was comparable in both series. When antibiotic sensitivity of microbial isolates of present study was compared to our previous report [17], there was no change in the sensitivity pattern in the last 10 years (Table 2). Since the GPC, GNB, and acid-fast organisms isolated in the cohort (from 2003 to 2012) were most commonly sensitive to ciprofloxacin, it could still remain the first choice of antibiotic in the management of scleral buckle infection until the microbiological validation.

The current study has the significant limitation of any retrospective study. Data such as the size of the buckle, position of Watzke sleeve, and types of peritomies were not analyzed. Number of eyes which underwent cataract surgey after SB surgery and before SB infection were also not analyzed, which has been reported to be an independent risk factor for SB infection [1,6,12,24]. We could not compare the incidence of buckle infection between silicone sponge and explant, as we did not have the information of total number of explant/sponge performed at our institute during the study period.

### Conclusions

In conclusion, scleral buckle infection is a rare complication of SB surgery which can present with varied clinical picture. Absence of suppuration or exposure of buckle does not exclude infection. There is no significant change in the microbiological profile and sensitivity patterns in the last decade; therefore, ciprofloxacin can still remain the treatment of choice in initial management of buckle infection. The results represent the experience in a single center in India, and the culture isolates might not be representative of or extrapolated to other parts of the world. Retinal detachment following buckle removal is uncommon and associated with favorable surgical outcome.

## Competing interests

The authors declare that they have no competing interests.

## Authors’ contributions

JC and AP facilitated the conception and design. JC, SN, AJ, SRM, and AP made the analysis and interpretation. JC, SN, AJ, AP, SJ, RRP, ANM, and HRW wrote the article. JC, AP, SJ, ANM, RRP, SRM, SS, TD, and HRW handled the critical revision of the article. JC, SN, AJ, AP, SJ, ANM, RRP, SRM, SS, TD, and HRW did the final approval of the article. SN and SRM led the data collection. JC and RRP took the provision of materials, patients, or resources. SN and ANM performed their statistical expertise. JC, SN, AJ, and AP carried the literature search. ANM, SJ, RRP, and TD gave administrative, technical, or logistic support. All authors read and approved the final manuscript.

## References

[B1] Ulrich RA Burton TC Infections following scleral buckling procedures Arch Ophthalmol 1974 3 213 215 10.1001/archopht.1974.01010010221007 4852420

[B2] Tsui I Scleral buckle removal: indications and outcomes Surv Ophthalmol 2012 3 253 263 10.1016/j.survophthal.2011.11.001 22516538

[B3] Farr AK Guyton DL Strabismus after retinal detachment surgery Curr Opin Ophthalmol 2000 3 207 210 10.1097/00055735-200006000-00010 10977229

[B4] Hilton GF Wallyn RH The removal of scleral buckles Arch Ophthalmol 1978 3 2061 2063 10.1001/archopht.1978.03910060449011 363106

[B5] Smiddy WE Miller D Flynn HW Jr Scleral buckle removal following retinal reattachment surgery: clinical and microbiologic aspects Ophthalmic Surg 1993 3 440 445 8351089

[B6] Covert DJ Wirostko WJ Han DP Lindgren KE Hammersley JA Connor TB Kim JE Risk factors for scleral buckle removal: a matched, case–control study Am J Ophthalmol 2008 3 434 439 10.1016/j.ajo.2008.05.023 18614132

[B7] Deokule S Reginald A Callear A Scleral explant removal: the last decade Eye (Lond) 2003 3 697 700 10.1038/sj.eye.6700491 12928679

[B8] Deutsch J Aggarwal RK Eagling EM Removal of scleral explant elements: a 10-year retrospective study Eye (Lond) 1992 3 570 573 10.1038/eye.1992.124 1289133

[B9] Lindsey PS Pierce LH Welch RB Removal of scleral buckling elements. Causes and complications Arch Ophthalmol 1983 3 570 573 10.1001/archopht.1983.01040010570007 6838415

[B10] Nguyen QD Lashkari K Hirose T Pruett RC McMeel JW Schepens CL Erosion and intrusion of silicone rubber scleral buckle Presentation and management Retina 2001 3 214 220 10.1097/00006982-200106000-00003 11421009

[B11] Roldan-Pallares M del Castillo Sanz JL Awad-El Susi S Refojo MF Long-term complications of silicone and hydrogel explants in retinal reattachment surgery Arch Ophthalmol 1999 3 197 201 10.1001/archopht.117.2.197 10037564

[B12] Schwartz PL Pruett RC Factors influencing retinal redetachment after removal of buckling elements Arch Ophthalmol 1977 3 804 807 10.1001/archopht.1977.04450050082007 871264

[B13] Wiznia RA Removal of solid silicone rubber exoplants after retinal detachment surgery Am J Ophthalmol 1983 3 495 497 6837692 10.1016/0002-9394(83)90270-2

[B14] Pastor JC Fernandez I Rodriguez de la Rua E Coco R Sanabria-Ruiz Colmenares MR Sanchez-Chicharro D Martinho R Ruiz Moreno JM Garcia Arumi J Suarez de Figueroa M Giraldo A Manzanas L Surgical outcomes for primary rhegmatogenous retinal detachments in phakic and pseudophakic patients: the Retina 1 Project–report 2 Br J Ophthalmol 2008 3 378 382 10.1136/bjo.2007.129437 18303159

[B15] Sun Q Sun T Xu Y Yang XL Xu X Wang BS Nishimura T Heimann H Primary vitrectomy versus scleral buckling for the treatment of rhegmatogenous retinal detachment: a meta-analysis of randomized controlled clinical trials Curr Eye Res 2012 3 492 499 10.3109/02713683.2012.663854 22577767

[B16] Joseph J Pathengay A Michael V Raju B Sharma S Das T In vitro efficacy of cefazolin and povidone-iodine 5% in eradicating microbial organisms adhered to broad scleral buckles Clin Experiment Ophthalmol 2006 3 390 391 10.1111/j.1442-9071.2006.01232.x 16764667

[B17] Pathengay A Karosekar S Raju B Sharma S Das T Hyderabad Endophthalmitis Research Group Microbiologic spectrum and susceptibility of isolates in scleral buckle infection in India Am J Ophthalmol 2004 3 663 664 10.1016/j.ajo.2004.04.056 15488804

[B18] Flindall RJ Norton EW Curtin VT Gass JD Reduction of extrusion and infection following episcleral silicone implants and cryopexy in retinal detachment surgery Am J Ophthalmol 1971 3 835 837 4324159 10.1016/0002-9394(71)90250-9

[B19] Lincoff H Nadel A O'Connor P The changing character of the infected scleral implant Arch Ophthalmol 1970 3 421 423 10.1001/archopht.1970.00990040423004 5492446

[B20] McMeel JW Naegele DF Pollalis S Badrinath SS Murphy PL Acute and subacute infections following scleral buckling operations Ophthalmology 1978 3 341 349 10.1016/S0161-6420(78)35660-8 662285

[B21] Holland SP Pulido JS Miller D Ellis B Alfonso E Scott M Costerton JW Biofilm and scleral buckle-associated infections. A mechanism for persistence Ophthalmology 1991 3 933 938 10.1016/S0161-6420(91)32199-7 1866148

[B22] Han DP Covert DJ Wirostko WJ Hammersley JA Lindgren KE Scleral buckle removal in the vitrectomy era: a 20-year clinical experience Retina 2013 3 387 391 10.1097/IAE.0b013e31826415d9 23064425

[B23] Wirostko WJ Covert DJ Han DP Connor TB Jr Kim JE Hammersley J Lindgren K Microbiological spectrum of organisms isolated from explanted scleral buckles Ophthalmic Surg Lasers Imaging 2009 3 201 202 10.3928/15428877-20090301-22 19320315

[B24] Covert DJ Wirostko WJ Han DP Lindgren KE Hammersley JA Connor TB Kim JE Risk factors for scleral buckle removal: a matched, case–control study Trans Am Ophthalmol Soc 2008 3 171 177 discussion 177–178 19277232 PMC2646451

